# Self-Reported Differences in Empowerment Between Lurkers and Posters in Online Patient Support Groups

**DOI:** 10.2196/jmir.992

**Published:** 2008-06-30

**Authors:** Cornelia F van Uden-Kraan, Constance HC Drossaert, Erik Taal, Erwin R Seydel, Mart AFJ van de Laar

**Affiliations:** ^2^Department of RheumatologyMedisch Spectrum TwenteEnschedeThe Netherlands; ^1^Institute for Behavioural ResearchUniversity of TwenteEnschedeThe Netherlands

**Keywords:** Online support groups, patients, lurkers, empowerment, breast cancer, fibromyalgia, arthritis

## Abstract

**Background:**

Patients who visit online support groups benefit in various ways. Results of our earlier study indicated that participation in online support groups had a profound effect on the participants’ feelings of “being empowered.” However, most studies of online patient support groups have focused on the members of these groups who actively contribute by sending postings (posters). Thus far, little is known about the impact for “lurkers” (ie, those who do not actively participate by sending postings).

**Objective:**

In the present study, we explored if lurkers in online patient support groups profit to the same extent as posters do.

**Methods:**

We searched the Internet with the search engine Google to identify all Dutch online support groups for patients with breast cancer, fibromyalgia, and arthritis. Invitations to complete an online survey were sent out by the owners of 19 groups. In the online questionnaire, we asked questions about demographic and health characteristics, use of and satisfaction with the online support group, empowering processes, and empowering outcomes. The online questionnaire was completed by 528 individuals, of which 109 (21%) identified themselves as lurkers.

**Results:**

Lurkers (mean age 47 years) were slightly older than active participants (mean age 43 years, *P* = .002), had a shorter disease history (time since diagnosis 3.7 years vs 5.4 years, *P* = .001), and reported lower mental well-being (SF 12 subscore 37.7 vs 40.5, *P* = .004). No significant differences were found in other demographic variables. Posters indicated visiting the online support groups significantly more often for social reasons, such as curiosity about how other members were doing, to enjoy themselves, as a part of their daily routine (all *P* < .001), and because other members expected them to be there (*P* = .003). Lurkers and posters did not differ in their information-related reasons for visiting the online support group. Lurkers were significantly less satisfied with the online support group compared to posters (*P* < .001). With regard to empowering processes such as “exchanging information” and “finding recognition,” lurkers scored significantly lower than posters. However, lurkers did not differ significantly from posters with regard to most empowering outcomes, such as “being better informed,” “feeling more confident in the relationship with their physician,” “improved acceptance of the disease,” “feeling more confident about the treatment,” “enhanced self-esteem,” and “increased optimism and control.” The exception was “enhanced social well-being,” which scored significantly lower for lurkers compared to posters (*P* < .001).

**Conclusion:**

Our study revealed that participation in an online support group had the same profound effect on lurkers’ self-reported feelings of being empowered in several areas as it had on posters. Apparently, reading in itself is sufficient to profit from participation in an online patient support group.

## Introduction

Studies have suggested that patients who use online support groups benefit in various ways [1-10]. Results of our earlier study indicated that participation in online support groups had a profound effect on the participants’ feelings of “being empowered” in several areas. Empowering outcomes mentioned by participants were being better informed; feeling confident with their physician, their treatment, and their social environment; improved acceptance of the disease; increased optimism and control; and enhanced self esteem and social well-being [11].

Most studies of online support groups for patients have focused on the members of these groups who actively contribute by sending postings (ie, posters). However, it is assumed that a considerable number of patients use online support groups in a passive way. Thus far, it is not known if those who do not actively participate by sending postings, the so-called lurkers, profit to the same extent from participating in online support groups as posters do.

 Although little is known about lurkers in online patient support groups, some studies have been conducted on lurkers in other online communities. Opinions about lurking and lurkers vary considerably. On the one hand, lurking is considered negative behavior. Smith and Kollock [12] describe lurkers as “free-riders”: they use the resources of online groups without giving back to them. Others consider lurking as acceptable and even beneficial. Many groups encourage lurking because in this way potential new users get a feeling for how the group operates and what kind of people participate in it [13,14]. Lurking can be desirable for very busy groups; if all subscribers to a group were to participate actively, it could cause repetition of queries and result in an overload of postings [15].

Study results indicated that lurking rates are highly variable: 0% to 99% [15-18]. Nonnecke and Preece [17] reported an average of 45.5% of lurkers in health-related online support groups.

Only a few studies have examined lurkers’ motives and experiences. In those studies, the participants were asked to indicate the reasons why they lurked instead of actually participating in the online communities. Reasons mentioned were concerns for privacy, no need to post, need to find out more about the group before participating, respect for others’ time and attention restrictions, no skills to make the software work, and no “click” with the group dynamics or a poor fit with the group [13,14,19,20]. Lurkers mostly indicated that they participated in an online group in order to receive information. In contrast, posters mentioned reasons such as to offer expertise, enjoy oneself, entertain others, build a professional relationship, tell stories, participate in conversations, make friends, get empathic support, and be a group member [13,14]. Nonnecke et al [14] also investigated possible differences in attitudes between lurkers and posters. Results showed that lurkers were less positive with regard to their online support group than those who post.

Although the above-mentioned studies provided us with valuable information concerning the characteristics of lurkers, little is known about the impact of lurking in online support groups [4]. Moreover, the previous studies focused on a wide range of online communities in which topics were discussed relating to the government, organizations, health, and e-commerce. It is unclear whether these results can be generalized to online patient support groups. In the present study, we therefore explored if lurkers in online patient support groups profit to the same extent as posters do. In addition, we explored to what extent lurking patients differed from posting patients with regard to demographic characteristics and usage and satisfaction with the online support group.

## Methods

### Sample and Procedure

We focused our study on online support groups for patients with breast cancer, fibromyalgia, and arthritis. We chose to explore these three groups because of the contrast among the illnesses (life-threatening, unexplained, and chronic disabling, respectively). We searched the Internet using the Google search engine to identify all Dutch online support groups for patients with breast cancer, fibromyalgia, and arthritis. In total, we found 20 groups. The online support groups differed in size and extent of activity; in the most active public support group under study, several hundred messages were exchanged daily, while in the least active support group, only 30 messages were exchanged during the last month. In total, 6 support groups under study were private groups to which we as researchers had no access. Therefore, we could not verify the number of messages exchanged in these groups.

Contact was established between the first author and the Web owners of the groups. The purpose of the study and methodology were explained to the Web owners. In addition, they were asked if they had any comments on the online questionnaire. We then asked the Web owners of these groups for permission to invite the participants to fill out our questionnaire. The Web owners of 19 groups (7 breast cancer, 6 fibromyalgia, and 6 arthritis) supported our study. In order not to intrude in the online support groups as researchers, we asked the Web owners of these 19 groups to send a posting in which participants were invited to fill out our online questionnaire. Criteria for inclusion were listed in the postings. The participants had to state that they had been diagnosed with breast cancer, fibromyalgia, or arthritis and had engaged passively or actively in online support groups. The medical diagnoses of the respondents were not verified with their physicians. Participants who were willing to fill out our questionnaire were invited to visit a Web page which provided information about our study and contact details of the first author. In total, 593 participants responded to our request. Obviously, a response percentage is not available due to the fact that it is not known how many patients participated in the online support groups under study. Of these participants, 65 filled in only the questions concerning their background and were thus not included in the data analysis, leaving 528 respondents. Of these respondents, 109 (21%) identified themselves as lurkers, which we defined in line with Preece et al [13] as “members who had never contributed a posting to an online group.”

### Instruments

#### Demographic and Health Characteristics

The respondents were asked to provide information about demographic characteristics such as age, sex, education, and diagnosis. Health-related quality of life was assessed with the SF 12, version 2. Standardized scores were calculated for physical and mental well-being, varying from 0 (poor well-being) to 100 (excellent well-being), with a mean of 50 and a standard deviation of 10 in the general population of the United States [21].

#### Use of and Satisfaction With the Online Support Group

Respondents were asked to indicate when they started visiting the online support group, how frequently they visited it, how long a visit lasted, and for what reasons they turned to the online support group. Lurking was measured with one single dichotomous item: “Did you ever contribute a posting to an online patient support group?”

The questionnaire also contained one item to measure general satisfaction with the online support group: “In general, how satisfied/dissatisfied are you with the online support group?” Respondents could answer on a 5-point scale that ranged from “very dissatisfied” (1) to “very satisfied” (5).

#### Empowering Processes

On the basis of the results of an earlier qualitative study [11], 29 items were formulated that described the empowering processes that took place in the online support groups. In all items, we asked for the frequency with which certain events happened in the online support group. Respondents could answer on a 4-point scale that ranged from “seldom or never” (1) to “often” (4). “Exchanging information” was measured with 9 items (alpha = .88). “Encountering emotional support” was measured with 12 items (alpha = .95), which was based on the Social Support List – Interaction [22]. “Finding recognition” was measured with 4 items (alpha = .70). “Helping others” was measured with 2 items (alpha = .82). Finally, “Sharing experiences” was measured with 2 items (alpha = .87).

#### Empowering Outcomes

On the basis of the results of an earlier qualitative study [11], 38 items were formulated that described empowering outcomes from participation in online support groups. All items had the format of a statement that began with “Through my participation in online support groups….” Respondents could answer on a 5-point scale that ranged from “completely disagree” (1) to “completely agree” (5). “Being better informed” was measured with 4 items (alpha = .85). “Feeling more confident in the relationship with their physician” was measured with 11 items (alpha = .91). “Improved acceptance of the illness” was measured with 5 items (alpha = .90). “Feeling more confident about the treatment” was measured with 5 items (alpha = .89). “Increased optimism and control over the future” was measured with 8 items (alpha = .76), partially based on the revised Illness Perception Questionnaire [23] and on the Dutch version of the Mastery Scale [24]. “Enhanced self-esteem” was measured with 3 items (alpha = .93), partially based on the Dutch version of the Rosenberg Self-Esteem Scale [25]. Finally, “Enhanced social well-being” was measured with 2 items (alpha = .70).

For an overview of the items belonging to all the above-mentioned constructs, see the Multimedia Appendix. For each construct, a mean total score was calculated.

### Data Analysis

The data were analyzed with the statistical software package SPSS 12.0 (SPSS Inc, Chicago, IL, USA). Differences in continuous variables between the posters and the lurkers were tested by means of Mann-Whitney U tests and differences in categorical variables by chi-square tests. In the data analysis, we excluded the respondents only if they were missing the data required for the specific analysis. Because of the great number of comparisons conducted, statistical significance was assumed when alpha < .01.

## Results

### Demographic and Health Characteristics of the Posters and Lurkers

Lurkers were somewhat older and were more recently diagnosed compared to posters (Table 1). No significant differences were found in sex, marital status, education, employment status, or type of diagnosis. Lurkers had a poorer mental well-being than posters. No significant differences between posters and lurkers were found in the physical component of the SF12.

**Table 1:** Demographics and health characteristics of posters and lurkers

	Posters^*^		Lurkers		*χ*^2^ (df)^†^	Mann-Whitney^‡^	*P*^§^
	No.	%	No.	%			
Sex					.000 (1)^||^		1.00
Female	392	94	102	94			
Male	27	6	7	6			
							
Age in years (posters: n = 416, lurkers: n = 109)						18291.50	.002
Mean (SD)	43 (10.4)		47 (9.9)				
Minimum	17		19				
Maximum	73		75				
							
Marital/relationship status					.094 (1)^||^		.76
Single	88	21	25	23			
In a relationship	331	79	84	77			
							
Education					2.24 (2)		.33
Low	129	32	42	39			
Middle	170	42	43	39			
High	111	27	24	22			
							
Employment status					1.33 (2)		.51
Paid job (> 20 hours)	128	31	39	36			
Paid job (≤ 20 hours)	54	13	11	10			
No job	234	56	59	54			
							
Diagnosis					.745 (3)		.86
Breast cancer	166	40	48	44			
Fibromyalgia	95	23	22	20			
Arthritis	97	23	24	22			
More diagnoses	61	15	15	14			
							
Time in years since diagnosis (posters: n = 385, lurkers: n = 96)						14382.50	.001
Mean (SD)	5.4 (6.1)		3.7 (4.6)				
Minimum	0		0				
Maximum	51		21				
					
Well-being (SF 12) (posters: n = 355, lurkers: n = 52)							
Physical well-being, mean (SD)	36.4 (11.6)		37.5 (9.9)			8294.50	.24
Mental well-being, mean (SD)	40.5 (6.4)		37.7 (5.8)			6960.50	.004

^*^No. is the number of respondents per item. Percentages are given with the total number of respondents per question as denominator (due to nonresponses, denominators may vary from question to question).

^†^Chi-square values are Pearson chi-square values with degrees of freedom in parentheses.

^‡^Mann-Whitney U value.

^§^ *P* value for chi-square tests and Mann-Whitney U tests comparing posters and lurkers.

^||^Chi-square values are Pearson chi-square values with continuity correction.

### Use of the Online Support Groups by Posters and Lurkers

The lurkers participated for a significantly shorter period of time compared to the posters (Table 2). Lurkers visited the online support groups significantly less frequently than the posters did. Most of the posters (64%) indicated that they visited the support group daily, compared to 27% of the lurkers. There was no significant difference between the posters and the lurkers concerning the duration of the visit to the online support group.

Lurkers and posters differed significantly with regard to some of the reasons for visiting the online support groups. Posters indicated visiting the online support groups significantly more often for social reasons, such as curiosity about how other members were doing, to enjoy themselves, as a part of their daily routine, and because other members expected them to be there. Lurkers and posters did not differ with their information-related reasons to visit the online support group.

In general, the lurkers were significantly less satisfied with the online support group compared to posters.

**Table 2:** Use of the online support group by posters and lurkers

	Posters^*^		Lurkers		*χ*^2^ (df)^†^	Mann-Whitney^‡^	*P*^§^
	No.	%	No.	%			
Number of years participating in an online support group (posters: n = 389, lurkers: n = 94)						13456.00	< .001
Mean (SD)	2.3 (2.1)		1.6 (2.1)				
Minimum	0		0				
Maximum	9		9				
							
Frequency of visits to online support group					75.756 (5)^||^		< .001
More than once during a day	140	35	6	7			
About one time during a day	121	30	18	20			
More than once in a week	96	24	28	31			
About one time in a week	31	8	19	21			
More than once in a month	0	0	0	0			
About once in a month	6	2	7	8			
Less than once in a month	6	2	12	13			
							
Duration of visits to online support group					3.560 (3)		.31
Less than 10 minutes	94	23	30	29			
10 minutes to 30 minutes	237	58	50	49			
30 minutes to 1 hour	57	14	18	18			
More than 1 hour	21	5	4	4			
							
Reasons for visiting the online support group							
Because I’m curious how other members are doing	244	58	34	31	24.298 (1)^¶^		< .001
It’s part of my daily routine	202	48	15	14	40.992 (1)^¶^		< .001
When I have a question about my disease	180	43	36	33	3.131 (1)^¶^		.08
To enjoy myself	157	38	15	14	21.070 (1)^¶^		< .001
When I heard new information about my illness	125	30	25	23	1.698 (1)^¶^		.19
When I have a lot of symptoms	92	22	20	18	.475 (1)^¶^		.49
When I feel lonely	92	22	15	14	3.106 (1)^¶^		.08
When I get new symptoms	106	25	27	25	.000 (1)^¶^		1.00
After visiting a doctor	61	15	6	6	5.609 (1)^¶^		.02
Before visiting a doctor	43	10	3	3	5.226 (1)^¶^		.02
Because other members expect me to be there	50	12	2	2	8.830 (1)^¶^		.003
					
General satisfaction with the online support group (posters: n = 375, lurkers: n = 63), mean (SD)	4.3 (0.79)		4.0 (0.65)			8652.50	< .001

^*^No. is the number of respondents per item. Percentages are given with the total number of respondents per question as denominator (due to nonresponses, denominators may vary from question to question).

^†^Chi-square values are Pearson chi-square values with degrees of freedom in parentheses.

^‡^Mann-Whitney U value.

^§^ *P* value for chi-square tests and Mann-Whitney U tests comparing posters and lurkers.

^||^The assumption of chi-square concerning the minimum expected cell frequency (5 or greater) has been violated. Therefore the answer option “more times a month” has been left out of this analysis.

^¶^Chi-square values are Pearson chi-square values with continuity correction.

### Empowering Processes

With regard to all empowering processes, lurkers scored significantly lower than the posters (Table 3). The processes that were reported the most frequently in the online support groups by both lurkers and posters were “exchanging information” and “finding recognition.”

**Table 3:** Mean scale scores processes for posters and lurkers

	Posters		Lurkers		Mann-Whitney^*^	*P*^†^
	No.	Mean (SD)	No.	Mean (SD)		
Exchanging information (1-4)	411	3.0 (0.54)	99	2.8 (0.59)	15560.00	< .001
Finding recognition (1-4)	387	2.9 (0.54)	73	2.5 (0.67)	9720.50	< .001
Sharing experiences (1-4)	387	2.8 (0.85)	73	1.9 (0.93)	6233.00	< .001
Encountering emotional support (1-4)	405	2.3 (0.74)	86	1.5 (0.61)	6272.50	< .001
Helping others (1-4)	387	2.3 (0.71)	73	1.6 (0.63)	6463.50	< .001

^*^Mann-Whitney U value.

^†^ *P* value for Mann-Whitney U tests comparing posters and lurkers.

### Empowering Outcomes

Table 4 shows that lurkers did not differ significantly from posters with regard to the empowering outcomes, with the exception of “enhanced social well-being.”

The lurkers experienced the outcome of “enhanced social well-being” significantly less often compared to the posters. The empowering outcome that was experienced to the strongest degree by both posters and lurkers was “being better informed.”

**Table 4:** Mean scale scores outcomes for posters and lurkers

	Posters		Lurkers		Mann-Whitney^*^	*P*^†^
	No.	Mean (SD)	No.	Mean (SD)		
Being better informed (1-5)	373	3.7 (0.76)	61	3.6 (0.66)	9403.50	.03
Enhanced social well-being (1-5)	359	3.4 (0.96)	52	2.8 (0.76)	5603.50	< .001
Feeling more confident in the relation with their physician (1-5)	369	3.4 (0.72)	58	3.3 (0.60)	10248.00	.60
Improved acceptance of the disease (1-5)	365	3.3 (0.91)	56	3.1 (0.92)	9001.50	.15
Feeling more confident about the treatment (1-5)	365	3.2 (0.79)	57	3.1 (0.79)	9112.50	.13
Enhanced self-esteem (1-5)	359	3.2 (0.94)	52	3.0 (0.83)	7790.00	.05
Increased optimism and control (1-5)	361	3.2 (0.59)	52	3.1 (0.64)	8268.00	.16

^*^Mann-Whitney U value.

^†^ *P* value for Mann-Whitney U tests comparing posters and lurkers.

## Discussion

### Principal Findings

To the best of our knowledge, this study is the first to empirically examine differences in perceived empowering outcomes between lurkers and posters. Our study revealed that, with the exception of the empowering outcome “enhanced social well-being,” participation in an online support group had the same profound effect on lurkers’ feelings of being empowered in several areas as it had on posters. Apparently, the mere reading of postings from others in online support groups can be beneficial for patients. Therefore, lurking in online support groups might be seen as a form of bibliotherapy. The idea of bibliotherapy is that well-being can be improved by reading self-help books or stories in which people can identify themselves with others [26]. Other studies have found evidence for online bibliotherapy; it has been shown to be effective in reducing depression [27], increasing self-management ability [26], and treating panic disorders [28].

Lurkers and posters did differ, however, with regard to the empowering outcome of “enhanced social well-being.” Fewer lurkers than posters reported that participating in an online support group led to a rise in their number of social contacts or to a decrease in loneliness.These results did not surprise us because it seems to be impossible to achieve new social contacts by lurking in an online support group.

In contrast to the empowering outcomes, we did find differences between lurkers and posters concerning the empowering processes executed in the online support groups. These differences not only appeared when focusing on processes that cannot be executed as a lurker, such as “helping others,” but also with processes such as “finding recognition.” These results are in line with the study results of Bane et al [29], who found indications in their study that lurkers in an online weight management group were less likely to see the group as a source of support and that it was less likely for them to find another group member with whom they could socially compare themselves.

An explanation for the significant difference between lurkers and posters with regard to the process “exchanging information” can, in our opinion, be linked to one of the frequently mentioned advantages of online support groups, namely that patients have the opportunity to request and receive informational support according to their personal needs and preferences [30]. Although lurkers have the option to read the information posted by others, they do not take advantage of the option to ask questions with specific concern for their own personal situation.

Our study indicated that lurkers were less satisfied than posters with the online support group. These results are in line with results of earlier studies that found that the majority of lurkers were significantly less enthusiastic than posters about the online group they participated in [13,14]. Nonnecke et al [14] suggested that lurking might even be a result of dissatisfaction with the online group, although they did mention that it is not clear whether lurking behavior causes dissatisfaction or whether dissatisfaction with the online group results in lurking.

This study also provided some insight into the demographic characteristics of lurkers in Dutch online support groups. The demographic populations of lurkers and posters were similar in this study with the exception of age. Lurkers were somewhat older compared to those who post. These results might indicate a relationship between a lack of computer skills and lurking since elderly people are in general less familiar with computers. In addition, one of the respondents to our questionnaire illustrated this problem: “I gave up trying to contribute a posting to [name of online support group]. I just cannot find out how to….”

Finally, the results of our study showed that lurkers are active for a significantly shorter period of time in the online support groups compared to the posters. This might indicate that among the lurkers, there are indeed new users of the online support groups who first want to get to know the group before they start posting. This phenomenon is referred to in the literature as “de-lurking” [20,31].

Several researchers have focused on methods to speed up the process of de-lurking, for example, by fostering receptive participation and by making the learning about the community and the first experiences as pleasant as possible [31] or by providing clear usability instructions [13]. The present study, however, indicates that for lurkers themselves it is not really necessary to de-lurk because they profit to the same extent from participating in online support groups as posters do. This does not mean that we encourage lurking. Lurking may be a problem for online patient support groups if there are few or no participants who contribute postings. According to Nonnecke et al [14], lurking is especially a problem for new online groups that do not yet have a sustainable group of active contributors. Silent online groups cannot survive because there is so much on offer on the Internet that people do not return to these groups [13].

In addition, this study showed that lurkers do not profit to the same extent as posters with regard to the outcome “enhanced social well-being” and that lurkers had a poorer mental well-being. These results might suggest that posting improves social or mental well-being. However, because we do not have baseline information about social and mental well-being at the time a patient joined an online support group, we cannot draw any conclusions about the causality of this relationship.

### Limitations of the Present Study

The findings of this study are limited by the relative small percentage of lurkers (21%) included. Although a response percentage is not available, we presume that the percentage of lurkers active in the online support groups under study is higher than 21% when we consider the study results of Nonnecke and Preece [17], who reported an average rate of 45.5% of lurkers in health-related online support groups.

In addition, a considerable number of participants only partially completed the questionnaire. To examine whether there was selective attrition, we compared those respondents who completed the questionnaire with the respondents who did not complete the questionnaire on crucial aspects, such as whether they were posters or lurkers (data not shown). This analysis showed that lurkers did not complete the questionnaire significantly more often than posters. Since the questions on empowering outcomes were at the end of the questionnaire, this might mean that those lurkers who did not feel empowered simply did not respond to the respective questions. However, we can also think of other viable reasons. According to Preece et al [13], lurkers usually do not have the inclination to respond to questionnaires. Therefore, it can also be expected that lurkers more often than posters decide not to complete a questionnaire. This might especially be the case if a questionnaire is of considerable length, such as the one used in our study.

Thus, the participants who chose to complete the online questionnaire are not necessarily representative of all lurkers and posters participating in online support groups for patients with breast cancer, fibromyalgia, and arthritis.

Finally, it should be taken into account that the results are self-perceived outcomes. Participants themselves estimated to what extent they profited from participation in online support groups. This does not prove that they truly profited from participation. Although this study provided us with relevant insights into the empowering outcomes as experienced by the posters and lurkers, a randomized controlled trial or a longitudinal study is required to evaluate whether posters and lurkers are truly empowered.

### Conclusion

Earlier studies showed that patients can profit from participation in online patient support groups. Our current study suggests that this not only applies to those patients who actively participate by sending postings, but also to those patients who only lurk in online patient support groups. Apparently, the use of online support groups, even if it consists of merely reading postings by others, might be beneficial for patients. Physicians should therefore acquaint their patients with the existence of online patient support groups since these groups offer the surplus value of patient expert information compared to regular medical information.

## Multimedia Appendix

Original Questionnaire (in Dutch)
