# Supplementary material for: Self-Reported Differences in Empowerment Between Lurkers and Posters in Online Patient Support Groups
Source: J Med Internet Res. 2008 Jun 30;10(2):e18. doi: 10.2196/jmir.992 (PMC2483925; doi:10.2196/jmir.992)
Supplement: Supplementary file 1 [file jmir_v10i2e18_app1.pdf]

APPENDIX TO:
van Uden-Kraan CF, Drossaert CHC, Taal E, Seydel ER, van de Laar MA
Self-Reported Differences in Empowerment Between Lurkers and Posters in Online Patient Support Groups
J Med Internet Res 2008;10(2):e18
<URL: http://www.jmir.org/2008/2/e18/> doi:10.2196/jmir.992

Appendix ‘Constructs empowering processes’

Answer categories: ‘seldom to never’; ‘sometimes’; ‘regularly’; ‘often’.

Exchanging information (α = .88):

The information and tips exchanged in this online support group are…

- … understandable
- … valuable
- … usable
- … new
- … applicable to my present situation
- … reliable
- … correct
- … of added value to the information I receive from my care providers
- … in line with the information I receive from my care providers

Exchanging social support (α = .95):

Does it ever happen in this online support group…

- … that someone in this online support group invites you to have (personal) contact outside this online support group?
- … that someone in this online support group starts a private conversation with you?
- … that someone in this online support group is empathic?
- … that someone in this online support group consoles you?
- … that someone in this online support group pays you a compliment?
- … that someone in this online support group is interested in you?
- … that someone in this online support group pays particular attention to you in special cases, such as during illness or moving house?
- … that someone in this online support group reassures you?
- … that someone in this online support group offers you sound advice?
- … that someone in this online support group points out your strengths?
- … that someone in this online support group confides in you?
- … that someone in this online support group asks you for your help or advice?

The comparison with other online support group members (α = .70):

Does it ever happen in this online support group that…

- … you recognize yourself in the stories of other online support group members?
- … you experience the sense of ‘not being the only one’?
- … others are an example to you?
- … you realize that you are not so bad off after all?

Helping others (α = .82):

Does it ever happen in this online support group that…

- … you can be an example to other participants?
- … you can offer advice and support to others?

APPENDIX TO:
van Uden-Kraan CF, Drossaert CHC, Taal E, Seydel ER, van de Laar MA
Self-Reported Differences in Empowerment Between Lurkers and Posters in Online Patient Support Groups
J Med Internet Res 2008;10(2):e18
<URL: http://www.jmir.org/2008/2/e18/> doi:10.2196/jmir.992

Sharing experiences ($\alpha = .87$):

Does it ever happen in this online support group that you can share …

- … your experiences with your illness with others?
- … your everyday experiences with others?

Appendix 'Constructs empowering outcomes'

Answer categories: 'completely disagree'; 'disagree'; 'neither agree nor disagree'; 'agree'; 'completely agree'.

Feel better informed (α = .85):
Through my participation in online support groups…
- … I feel better informed as a patient.
- … I understand my illness better.
- … I have a clearer picture about my illness.
- … I feel like I have more (correct) knowledge at my disposal to deal better with my illness.

Feeling more confident in the relationship with their physician (α = .91):
Through my participation in online support groups…
- … I feel better prepared for a doctor's appointment.
- … I am more knowledgeable about which questions to ask my physician.
- … I can explain my needs to my physician better.
- … I have more courage to raise matters with my physician.
- … I am more able to oppose my physician.
- … I understand the information provided by my physician better.
- … the relationship with my physician has improved.
- … the relationship with my physician has deteriorated.
- … I am more able to judge when I really need the help of my physician.
- … I feel less dependent on my physician.
- … I am more able to think along with my physician about my treatment.

Improved acceptance of the illness (α = .90):
Through my participation in online support groups…
- … I am able to be more open about my own illness.
- … I can tell others more easily when I am no longer able to do something.
- … I can ask others for help more quickly.
- … I can give in to my illness better.
- … I can accept my illness better.

Increased confidence in their treatment (α = .89):
Through my participation in online support groups…
- … I can stick to my treatment regime better.
- … I am more able to follow the medical guidelines and advice of my physician.
- … I know where to go with questions about my illness.
- … I feel I am more skilled at dealing well with my illness.
- … I feel able to make the right decisions with regard to my illness.

Increased optimism and control over the future (α = .76):

Through my participation in online support groups…

- … I feel more in charge of the course of my illness.
- … I feel I can influence my illness myself.
- … I feel more in control over what is happening to me.
- … I feel less in control over what is happening to me.
- … I feel that what happens to me in the future is to a large degree dependent on myself.
- … I have learned to be more positive.
- … I have more faith in the future.
- … I have less faith in the future.

Enhanced self-esteem (α = .93):
Through my participation in online support groups…

- … I have a greater sense of worth.
- … I have a more positive attitude towards myself.
- … I am in general more content with myself.

Enhanced social well-being (α = .70):
Through my participation in online support groups…

- … I feel less lonely.
- … I have made new social contacts.

APPENDIX TO:
van Uden-Kraan CF, Drossaert CHC, Taal E, Seydel ER, van de Laar MA
Self-Reported Differences in Empowerment Between Lurkers and Posters in Online Patient Support Groups
J Med Internet Res 2008;10(2):e18
<URL: http://www.jmir.org/2008/2/e18/> doi:10.2196/jmir.992

Vragenlijst “Online Lotgenotencontact”

Beste deelnemer aan [naam forum],

Deze vragenlijst maakt deel uit van een onderzoek door de Universiteit Twente naar de betekenis van lotgenotencontact via internet fora voor patiënten met borstkanker, reuma en fibromyalgie.

De vragenlijst bestaat uit de volgende drie onderdelen:

- Wie bent u en hoe zou u uw gebruik van internet fora beschrijven?
- Hoe ervaart u uw deelname aan internet fora?
- In hoeverre bent u veranderd door uw deelname aan internet fora?

Bij elke vraag is het de bedoeling dat u een antwoord kiest, dat zo goed mogelijk overeen komt met uw mening. Na het invullen van de vragen, kunt u onderaan op 'verder' klikken. Zo loopt u vanzelf door de vragenlijst.

Uw antwoorden worden uiteraard volstrekt anoniem verwerkt.

Voor meer informatie over dit onderzoek kunt u contact opnemen met drs. Nelly van Uden-Kraan (c.f.vanuden-kraan@utwente.nl).

Alvast hartelijk dank voor uw medewerking!

Deel 1:
Wie bent u en hoe zou u uw gebruik van internet fora beschrijven?

Het eerste gedeelte van de vragenlijst bestaat uit een aantal vragen over u zelf en over uw gebruik van internet fora.

1. Wat is uw geslacht?

☐ Man
☐ Vrouw

2. Hoe oud bent u?

…… jaar

3. Wat is uw burgerlijke staat?

☐ Alleenstaand
☐ Gehuwd / geregistreerd partnerschap
☐ Duurzaam samenwonend
☐ Gescheiden
☐ Weduwe / weduwnaar

4. Wat is uw hoogst genoten opleiding?

☐ Lagere school/Basisschool
☐ Lager beroepsonderwijs/Huishoudschool
☐ VMBO/Mavo/Mulo/Ulo
☐ Middelbaar beroepsonderwijs (MBO)
☐ Havo/MMS
☐ VWO/Gymnasium/HBS
☐ Hoger beroepsonderwijs (HBO)
☐ Universiteit
☐ Anders, namelijk ……………………………………………………………………

5. Wat is de beste beschrijving van uw arbeidssituatie?

☐ Betaald werk (meer dan 20 uur per week)
☐ Betaald werk (minder dan of gelijk aan 20 uur per week)
☐ Huisman/Huisvrouw
☐ Vrijwilligerswerk
☐ Student/Scholier
☐ Ziektewet
☐ WAO/WIA
☐ (Pré-)pensioen
☐ Reïntegratietraject
☐ Werkloos
☐ Anders, namelijk ……………………………………………………………………

6. In welke rol bezoekt u internetfora?

☐ als patiënt
☐ als vermoedelijke patiënt
☐ als partner van een patiënt (exit)
☐ als familielid van een patiënt (exit)
☐ als kennis van een patiënt (exit)
☐ als zorgverlener (exit)
☐ als adverteerder (exit)
☐ als onderzoeker (exit)
☐ als moderator en tevens patiënt
☐ als moderator en geen patiënt (exit)

7. Welke ziekte is bij u geconstateerd? (Het is de bedoeling dat u bij deze vraag één antwoord kiest: als u bijvoorbeeld meerdere vormen van reuma heeft graag alleen de voor u belangrijkste aankruisen.)

□ Borstkanker

□ Fibromyalgie (chronische diffuse pijn en vermoeidheid)

□ Reumatoïde artritis
□ Artrose
□ Artritis psoriatica
□ Polymyalgia reumatica
□ Bechterew
□ S.L.E.
□ Sclerodermie (systemische sclerose)
□ Syndroom van Sjögren
□ Jicht
□ Lage rugpijn
□ Tendinitis/Bursitis
□ Osteoporose

□ Anders, namelijk …………………………………………………………………………

8. Wanneer werd bij u deze diagnose gesteld? (Mocht u zich de exacte maand niet meer herinneren dan kunt u voldoen met het invullen van het jaartal)

………… (maand) ………… (jaar)

□ De diagnose is nog niet gesteld

9. Sinds wanneer neemt u deel aan internet fora in relatie tot uw ziekte? (Mocht u zich de exacte maand niet meer herinneren dan kunt u voldoen met het invullen van het jaartal)

………… (maand) ………… (jaar)

APPENDIX TO:
van Uden-Kraan CF, Drossaert CHC, Taal E, Seydel ER, van de Laar MA
Self-Reported Differences in Empowerment Between Lurkers and Posters in Online Patient Support Groups
J Med Internet Res 2008;10(2):e18
<URL: http://www.jmir.org/2008/2/e18/> doi:10.2196/jmir.992

Invulinstructie

De hierna volgende vragen gaan over het gebruik van het internetforum dat u het meest bezoekt in relatie tot uw ziekte.

10. Hoe bent u bij dit internet forum terecht gekomen?

☐ Tijdens het surfen op internet
☐ Tijdens het zoeken naar informatie over mijn ziekte op internet
☐ Via een ander forum
☐ Er ergens over gelezen
☐ Via een vriend/familielid/bekende
☐ Via de patiëntenvereniging
☐ Via een andere patiënt
☐ Er op gewezen door mijn arts en/of een andere zorgverlener
☐ Dit forum is door mezelf opgericht
☐ Anders, namelijk ……………………………………………………………………………………………

11. Hoe vaak bezoekt u dit internet forum gemiddeld?

☐ Meerdere keren per dag
☐ Ongeveer één keer per dag
☐ Meerdere keren per week
☐ Ongeveer één keer per week
☐ Meerdere keren per maand
☐ Ongeveer één keer in de maand
☐ Minder dan één keer in de maand
☐ Anders, namelijk ……………………………………………………………………………………………

12. Wat vormt voor u een aanleiding om dit forum te bezoeken? (meerdere antwoorden mogelijk)

☐ Het maakt onderdeel uit van mijn dagelijkse routine
☐ Voor een bezoek aan mijn arts
☐ Na een bezoek aan mijn arts
☐ Op het moment dat ik veel klachten heb
☐ Op het moment dat ik te maken krijg met nieuwe klachten
☐ Als ik een vraag heb over mijn ziekte
☐ Als ik me eenzaam voel
☐ Als ik nieuwe informatie met betrekking tot mijn ziekte heb gehoord
☐ Voor de gezelligheid
☐ Als ik nieuwsgierig ben hoe het met andere leden is
☐ Als de andere groepsleden rekenen op mijn aanwezigheid
☐ Anders, namelijk ……………………………………………………………………………………………

13. Hoe lang duurt uw bezoek aan dit forum gemiddeld per keer?

☐ Minder dan 10 minuten
☐ 10 tot 30 minuten
☐ 30 minuten tot één uur
☐ Langer dan één uur
☐ Anders, namelijk ……………………………………………………………………………………………………

14. Heeft u wel eens berichten op dit forum geplaatst?

☐ Nee, ik heb nog nooit een bericht geplaatst
☐ Ja

15. Hoe vaak heeft u in de afgelopen vier weken een bericht op het forum geplaatst?

☐ Nooit
☐ Enkele berichten, maar minder dan één keer per week
☐ Één keer per week
☐ Meerdere keren per week, maar niet elke dag
☐ Elke dag één bericht
☐ Elke dag meerdere berichten

Deel 2:

Hoe ervaart u uw deelname aan het internet forum waar u het meest actief bent?

In het tweede gedeelte van deze vragenlijst willen we graag achterhalen hoe u uw deelname aan het internet forum waar u het meest actief bent, ervaart.

16. De informatie en tips die worden uitgewisseld op dit forum zijn…

1 = zelden of nooit
2 = soms
3 = regelmatig
4 = meestal

a. … begrijpelijk
b. … waardevol
c. … bruikbaar
d. … nieuw
e. … van toepassing op mijn persoonlijke situatie
f. … betrouwbaar
g. … juist
h. … van toegevoegde waarde op de informatie die ik van mijn zorgverleners ontvang
i. … in overeenstemming met de informatie die ik van mijn zorgverleners ontvang

17. Gebeurt het wel eens dat men via dit forum…

1 = zelden of nooit
2 = soms
3 = regelmatig
4 = vaak

a. … u uitnodigt voor persoonlijk contact buiten dit forum om?
b. … gezellig met u een persoonlijk praatje maakt?
c. … genegenheid voor u toont?
d. … u troost?
e. … u complimenten geeft?
f. … interesse voor u toont?
g. … u extra aandacht biedt in bijzondere gevallen, zoals een ziekte of een verhuizing?
h. … u geruststelt?
i. … u goede raad geeft?
j. … uw sterke punten naar voren haalt?
k. … u in vertrouwen neemt?
l. … u om hulp of advies vraagt?

18. Gebeurt het wel eens op dit forum dat…

1 = zelden of nooit
2 = soms
3 = regelmatig
4 = vaak

a. … u zich herkent in de verhalen van andere forumgenoten?
b. … u het “ik ben niet de enige gevoel” ervaart?
c. … anderen een voorbeeld voor u zijn?
d. … u beseft dat het met u zo slecht nog niet gaat?
e. … u andere deelnemers tot een voorbeeld kan zijn?
f. … u andere deelnemers van advies en steun kan voorzien?
g. … u uw ervaringen met uw ziekte kan delen met anderen?
h. … u uw alledaagse ervaringen kan delen met anderen?

19. Hoe tevreden of ontevreden bent u met dit forum?

☐ Zeer tevreden
☐ Tevreden
☐ Niet tevreden / niet ontevreden
☐ Ontevreden
☐ Zeer ontevreden

APPENDIX TO:
van Uden-Kraan CF, Drossaert CHC, Taal E, Seydel ER, van de Laar MA
Self-Reported Differences in Empowerment Between Lurkers and Posters in Online Patient Support Groups
J Med Internet Res 2008;10(2):e18
<URL: http://www.jmir.org/2008/2/e18/> doi:10.2196/jmir.992

Deel 3:

In hoeverre bent u veranderd door uw deelname aan internet fora?

In het derde gedeelte van deze vragenlijst willen we graag achterhalen in hoeverre u bent veranderd door uw deelname aan internetfora.

20. Geef hieronder aan in hoeverre u het eens bent met onderstaande stellingen.

1 = helemaal mee oneens
2 = mee oneens
3 = niet mee eens / niet mee oneens
4 = mee eens
5 = helemaal mee eens

Door mijn deelname aan internetfora…

a. … voel ik mij een beter geïnformeerde patiënt.
b. … begrijp ik mijn ziekte beter.
c. … heb ik een duidelijker beeld van mijn ziekte.

21. Geef hieronder aan in hoeverre u het eens bent met onderstaande stellingen.

1 = helemaal mee oneens
2 = mee oneens
3 = niet mee eens / niet mee oneens
4 = mee eens
5 = helemaal mee eens

!NB: Met arts wordt in principe de medisch specialist bedoeld waarmee u het meest frequent contact mee heeft. Mocht u niet onder behandeling van een medisch specialist staan, dan kan u uitgaan van uw huisarts.

Door mijn deelname aan internetfora…

a. … voel ik mij beter voorbereid op een consult met mijn arts.
b. … weet ik beter welke vragen ik aan mijn arts moet stellen.
c. … weet ik beter hoe ik mijn behoeften aan mijn arts moet duidelijk maken.
d. … durf ik zaken beter aan te kaarten bij mijn arts.
e. … durf ik meer tegengas te geven aan mijn arts.
f. … begrijp ik de informatie die ik van mijn arts krijg beter.
g. … is de verstandhouding met mijn arts verbeterd.
h. … is de verstandhouding met mijn arts verslechterd.
i. … kan ik beter inschatten wanneer ik echt de hulp van mijn arts nodig heb.
j. … voel ik me minder afhankelijk van mijn arts.
k. … kan ik beter meedenken met mijn arts over mijn behandeling.

APPENDIX TO:
van Uden-Kraan CF, Drossaert CHC, Taal E, Seydel ER, van de Laar MA
Self-Reported Differences in Empowerment Between Lurkers and Posters in Online Patient Support Groups
J Med Internet Res 2008;10(2):e18
<URL: http://www.jmir.org/2008/2/e18/> doi:10.2196/jmir.992

22. Geef hieronder aan in hoeverre u het eens bent met onderstaande stellingen.

1 = helemaal mee oneens
2 = mee oneens
3 = niet mee eens / niet mee oneens
4 = mee eens
5 = helemaal mee eens

Door mijn deelname aan internetfora…

a. … lukt het mij beter om mij aan mijn behandelvoorschriften te houden.
b. … ben ik beter in staat om de medische richtlijnen en adviezen van mijn arts op te volgen.
c. … heb ik meer het gevoel over de juiste kennis te beschikken om goed met mijn ziekte om te kunnen gaan.
d. … weet ik waar ik terecht kan met vragen over mijn ziekte.
e. … heb ik meer het gevoel over de juiste vaardigheden te beschikken om goed met mijn ziekte om te kunnen gaan.
f. … heb ik het gevoel de juiste beslissingen ten aanzien van mijn ziekte te kunnen maken.

23. Geef hieronder aan in hoeverre u het eens bent met onderstaande stellingen.

1 = helemaal mee oneens
2 = mee oneens
3 = niet mee eens / niet mee oneens
4 = mee eens
5 = helemaal mee eens

Door mijn deelname aan internetfora…

a. … durf ik opener te zijn over mijn eigen ziekte.
b. … durf ik beter tegen mijn omgeving te zeggen als het niet meer gaat.
c. … durf ik mijn omgeving sneller om hulp te vragen.
d. … durf ik beter toe te geven aan mijn ziekte.
e. … kan ik mijn ziekte beter accepteren.

24. Geef hieronder aan of u het eens bent met onderstaande stellingen.

1 = helemaal mee oneens
2 = mee oneens
3 = niet mee eens / niet mee oneens
4 = mee eens
5 = helemaal mee eens

Door mijn deelname aan internetfora…

a. … heb ik het gevoel het verloop van mijn ziekte meer in eigen hand te hebben.
b. … heb ik meer het gevoel dat ik zelf mijn ziekte kan beïnvloeden.
c. … heb ik het gevoel meer controle te hebben over de dingen die met mij gebeuren.
d. … heb ik het gevoel minder controle te hebben over de dingen die met mij gebeuren.
e. … heb ik meer het gevoel dat wat er in de toekomst met mij gebeurt in grote mate van mezelf afhangt.
f. … heb ik meer vertrouwen gekregen in de toekomst.
g. … heb ik minder vertrouwen gekregen in de toekomst.
h. … heb ik geleerd positiever in het leven te staan.

25. Geef hieronder aan in hoeverre u het eens bent met onderstaande stellingen.

1 = helemaal mee oneens
2 = mee oneens
3 = niet mee eens / niet mee oneens
4 = mee eens
5 = helemaal mee eens

Door mijn deelname aan internetfora…

a. … voel ik me een waardevoller persoon.
b. … neem ik een positievere houding aan tegenover mezelf.
c. … ben ik over het algemeen meer tevreden over mezelf.
d. … voel ik me minder eenzaam.
e. … heb ik nieuwe sociale contacten opgedaan.

Tot slot

De laatste vragen gaan over uw standpunten t.a.v. uw gezondheid.

26. Hoe zou u over het algemeen uw gezondheid noemen?

☐ Uitstekend
☐ Zeer goed
☐ Goed
☐ Matig
☐ Slecht

27. De volgende vragen gaan over bezigheden die u misschien doet op een doorsnee dag. Wordt u door uw gezondheid op dit moment beperkt bij deze bezigheden?

1 = ja, ernstig beperkt
2 = ja, een beetje beperkt
3 = nee, helemaal niet beperkt

a. Matige inspanning, zoals een tafel verplaatsen, stofzuigen, zwemmen of fietsen
b. Een paar trappen lopen

28. Hoe vaak heeft u in de afgelopen 4 weken, één van de volgende problemen bij uw werk of andere dagelijkse bezigheden gehad, ten gevolge van uw lichamelijke gezondheid?

1 = altijd
2 = meestal
3 = soms
4 = zelden
5 = nooit

a. U heeft minder bereikt dan u zou willen
b. U was beperkt in het soort werk of andere bezigheden

29. Hoe vaak heeft u in de afgelopen 4 weken, één van de volgende problemen ondervonden bij uw werk of andere dagelijkse bezigheden, ten gevolge van emotionele problemen (zoals depressieve of angstige gevoelens)?

1 = altijd
2 = meestal
3 = soms
4 = zelden
5 = nooit

a. U heeft minder bereikt dan u zou willen
b. U deed uw werk of andere bezigheden niet zo zorgvuldig als gewoonlijk

30. In welke mate bent u de afgelopen 4 weken door pijn gehinderd in uw normale werk (zowel werk buitenshuis als huishoudelijk werk)?

☐ Helemaal niet
☐ Een klein beetje
☐ Nogal
☐ Veel
☐ Heel erg veel

31. Deze vragen gaan over hoe u zich voelt en hoe het met u ging in de afgelopen 4 weken. Wilt u alstublieft bij elke vraag het antwoord geven dat het best benadert hoe u zich voelde?

1 = altijd
2 = meestal
3 = soms
4 = zelden
5 = nooit

Hoe vaak gedurende de afgelopen 4 weken…

a. Voelde u zich rustig en tevreden?
b. Had u veel energie?
c. Voelde u zich somber en neerslachtig?

32. Hoe vaak hebben u lichamelijke gezondheid of emotionele problemen u gedurende de afgelopen 4 weken gehinderd bij uw sociale activiteiten (zoals vrienden of familie bezoeken, etc.)?

☐ Altijd
☐ Meestal
☐ Soms
☐ Zelden
☐ Nooit

Einde van de vragenlijst

U bent aan het einde gekomen van de vragenlijst. Hartelijk bedankt voor het invullen! Mocht u nog opmerkingen hebben, dan kunt u die hieronder kwijt.

…

Klikt u op versturen om de vragenlijst af te sluiten en uw antwoorden te verzenden.
